# Vat Photopolymerization of Cemented Carbide Specimen

**DOI:** 10.3390/ma14247631

**Published:** 2021-12-11

**Authors:** Thomas Rieger, Tim Schubert, Julian Schurr, Andreas Kopp, Michael Schwenkel, Dirk Sellmer, Alexander Wolff, Juliane Meese-Marktscheffel, Timo Bernthaler, Gerhard Schneider

**Affiliations:** 1 Materials Research Institute, Faculty Mechanical Engineering and Materials Science, Aalen University, 73430 Aalen, Germany; Tim.Schubert@hs-aalen.de (T.S.); Julian.Schurr@hs-aalen.de (J.S.); Andreas.Kopp@hs-aalen.de (A.K.); Michael.Schwenkel@hs-aalen.de (M.S.); Timo.Bernthaler@hs-aalen.de (T.B.); Gerhard.Schneider@hs-aalen.de (G.S.); 2 Department of Mechanical Engineering, Karlsruhe Institute of Technology, 76131 Karlsruhe, Germany; 3 MAPAL Precision Tool Dr. Kress KG, 73431 Aalen, Germany; Dirk.Sellmer@mapal.com; 4 H.C. Starck Tungsten GmbH, 38642 Goslar, Germany; Alexander.Wolff@hcstarck.com (A.W.); Julia.Meese-Marktscheffel@hcstarck.com (J.M.-M.)

**Keywords:** cemented carbide, additive manufacturing, vat photopolymerization, characterization, heat treatment

## Abstract

Numerous studies show that vat photopolymerization enables near-net-shape printing of ceramics and plastics with complex geometries. In this study, vat photopolymerization was investigated for cemented carbide specimens. Custom-developed photosensitive WC-12 Co (wt%) slurries were used for printing green bodies. The samples were examined for defects using quantitative microstructure analysis. A thermogravimetric analysis was performed to develop a debinding program for the green bodies. After sintering, the microstructure and surface roughness were evaluated. As mechanical parameters, Vickers hardness and Palmqvist fracture toughness were considered. A linear shrinkage of 26–27% was determined. The remaining porosity fraction was 9.0%. No free graphite formation, and almost no η-phase formation occurred. WC grain growth was observed. 76% of the WC grains measured were in the suitable size range for metal cutting tool applications. A hardness of 1157 HV10 and a Palmqvist fracture toughness of 12 $\mathrm{MPa}\sqrt{m}$ was achieved. The achieved microstructure exhibits a high porosity fraction and local cracks. As a result, vat photopolymerization can become an alternative forming method for cemented carbide components if the amount of residual porosity and defects can be reduced.

## 1. Introduction

**Figure 1:**
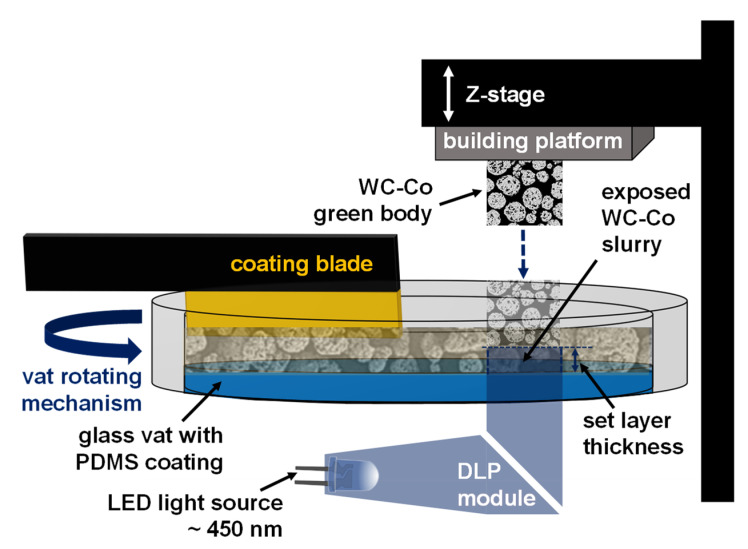
Schematic drawing of vat photopolymerization.

Cemented carbides are mainly used in the manufacturing industry for cutting, grinding, and drilling applications. These materials are characterized by high strength and wear resistance, especially at high temperatures and in corrosive environments [1]. A variety of material properties can be achieved through a defined fraction of fine refractory carbides in a ductile metallic matrix [2]. Using tungsten carbide (WC) (the hard material phase) and cobalt (Co) (the metallic binder phase) is the preferred material system that combines high strength with high fracture toughness [3]. Tools made of cemented carbides are usually manufactured through pressing and extrusion with subsequent sintering [4]. Also, metal powder injection molding (MIM) can be used to manufacture cemented carbide components [5]. Pressing and the MIM process require a specific and expensive tool for each individual component, which makes the manufacturing of small batches only rarely economically attractive. Post-processing has limited applicability and is expensive for cemented carbide components. Additive manufacturing (AM) is a fast, cost-effective, and resource-saving method for the customized production of small batches and components with complex geometries. Unused material can be reused for printing new components [6]. New tool designs can be realized based on the layer by layer technology. Inner cooling structures can be integrated with optimized design, shape, and diameter [7]. These new cooling structures can enable work at higher cutting speeds based on more efficient cooling, increasing the tool’s lifetime [8]. Vat photopolymerization provides a new method for forming cemented carbide components based on creating a green body out of a photosensitive slurry with subsequent debinding and sintering. During the building process, the slurry is homogeneously distributed over a polydimethylsiloxane (PDMS)-covered glass vat. Afterwards, the building platform is immersed in the slurry to the set layer thickness. A digital light processing (DLP) module is used to polymerize the cross-section of the component by light exposure [9]. The excess slurry can be removed from the vat after printing and prepared for the next print job by using a stirrer. Figure 1 shows a schematic drawing of the vat photopolymerization.

Subsequently, the component is cleaned of uncured slurry using a solvent and compressed air. This vat photopolymerization method is mainly used for ceramics and plastics [10]. Compared to powder bed fusion, vat photopolymerization requires no powder flowability. Compared to binder jetting, material jetting, and material extrusion, the area of the building platform can be cured in one step and does not require a local deposition of the slurry or organic binder. Furthermore, no complex filament manufacturing is necessary as required for material extrusion. However, it is worth mentioning that binder jetting, material jetting, and material extrusion have already been used to manufacture cemented carbide specimens with properties similar to those of conventionally pressed and sintered components [11,12,13,14,15]. A challenge of metal and ceramic vat photopolymerization is that the particles show a critical influence on the curing behavior of the slurry. Particles lead to scattering and absorption effects during exposure [16]. Dark particles especially show a strong absorption behavior in the wavelength range between 400 and 500 nm [17]. The light required to cure the photosensitive resin is strongly absorbed in these dark particles, and the achievable curing depth is limited [18,19]. Early tests have already shown that vat photopolymerization can also be used to work with cemented carbide slurries [20]. Photosensitive slurries with 40 vol% of WC and Co particles were developed and adapted with dispersants to increase the volume fraction of particles along with a thixotropic agent to prevent sedimentation effects [21]. Thixotropic agents support the stability due to a reversible reduction of viscosity during the coating operation and a subsequent increase of viscosity afterward [22]. A high volume fraction of particles is required to enable uniform shrinkage during heat treatment [23,24]. Inhomogeneities lead to visible defects on the surface, increased porosity, distortions, and cracks. Defects lead to reduced strength and ductility properties of the sintered component [24,25,26]. Debinding of the manufactured green bodies is one of the most critical process steps. The absence of open pores and a high polymer fraction as a binder makes thermal debinding very critical. In the initial state, defects such as cracks or bloating can occur due to stresses formed by trapped gas during the decomposition of the polymer. The component’s strength decreases first due to the polymer’s thermal softening and afterwards due to the loss of polymer. Stresses also act on the part, leading to cracks or deformation when the polymer degrades. To prevent the formation of defects, it is a common practice to use prolonged heating rates [27]. After debinding, cemented carbides are heat treated by liquid phase sintering. During heating, solid-state sintering starts. Dissolution and transport of the material take place by solid state diffusion and bulk transport [28]. After the melt is formed, the liquid spreads between the solid grains, breaking the sinter bonds and inducing grain rearrangement. Densification takes place due to melting of the liquid phase, wetting, and rearrangement of the particles. In the further sintering stage, densification occurs due to solution and re-precipitation [29]. Preferably small WC grains dissolve in the cobalt binder phase. Tungsten and carbon dissolved in the cobalt binder phase begin to re-precipitate on undissolved WC grains and grow at the expense of smaller grains (Ostwald ripening) [28]. In the next sintering stage, a slow densification occurs in which the liquid phase occupies the intergranular regions between the grains. Due to the solubility of the solid phase in the liquid phase, compaction is combined with solution and precipitation until the final density is reached. Further grain growth takes place even after full density is reached [30]. During subsequent cooling, further precipitation occurs and the binder phase becomes solid again [31]. For cemented carbides, the carbon balance during the sintering process determines the formation of the microstructure. If decarburization occurs during the process, a sub-stoichiometric W-Co carbide phase, called the η-phase, is formed [31,32]. This brittle phase negatively influences the fracture toughness of cemented carbides. If the carbon content is too high, free carbon is present in the form of graphite, which also negatively influences the mechanical material properties. Therefore, the carbon content during heat treatment should be kept within narrow limits where neither η-phase nor free graphite occur [31,33].

In this work, a custom-developed WC-12 Co (wt%) slurry was prepared to manufacture cemented carbide green bodies. A debinding concept was investigated using thermogravimetric analysis. The green bodies and the final sintered specimens were evaluated using light microscopy (LM) and scanning electron microscopy (SEM), combined with machine-learning tools and grayscale threshold segmentation for quantitative microstructure analysis and X-ray diffraction (XRD) analysis. The surface roughness after sintering was determined by confocal microscopy (CM). As mechanical parameters, hardness and Palmqvist fracture toughness were determined and compared with conventionally manufactured cemented carbide properties.

## 2. Materials and Methods

### 2.1. Characterization of Raw Materials

Spray-dried and sintered WC–Co granules (88:12 wt%) with an apparent density of 4.93 g/cm^3^ were used as the starting material. The granule size distribution was investigated using laser diffraction (HELOS H4299, Sympatec GmbH, Clausthal-Zellerfeld, Germany). Scanning electron microscopy (Sigma 300 VP, Carl Zeiss Microscopy GmbH, Oberkochen, Germany) was used to determine the morphology and the inner structure of the granules. For morphology analysis, a powder sample was homogeneously distributed on a SEM sample holder using the Nebula Particle Disperser (Thermo Fisher Scientific, Dreieich, Germany). The WC particle FERET_max_ distribution, inner porosity fraction and the chemical composition of the granules were investigated in cross-section SEM images. Quantitative microstructure analysis in combination with machine-learning based pixel segmentation (ZEN core 3.2, Carl Zeiss Microscopy GmbH, Oberkochen, Germany) was applied to quantify the characteristics of the granules. To evaluate the WC particle FERET_max_ distribution, >1000 WC particles were evaluated. A total of 20 g of the WC–Co granules were used for the multi-point BET (Brunauer–Emmett–Teller) surface area measurement using nitrogen adsorption at 77.4 K (Quantachrome NOVA4200e, Anton Paar QuantaTec Inc., Boynton Beach, FL, USA). The samples were degassed for 1 h at 200 °C under vacuum before measurement.

### 2.2. Slurry Preparation for Vat Photopolymerization

The monomer 1,6-Hexanediol (2EO) diacrylate (HDEODA) (IGM Resins B.V., Waalwijk, The Netherlands) was chosen as the main monomer because of its low viscosity and Di(trimethylolpropane) tetraacrylate (DiTMPTA) (Sigma Aldrich Chemie GmbH, Taufkirchen, Germany) for its cross-linking ability. The monomer and cross-linker were mixed in a mass ratio of 6:1. Subsequently, 1 wt% of the rheological additive RHEOBYK-410 (BYK-Chemie GmbH, Wesel, Germany) was added to the mixture, based on the total slurry weight. The agent shows thixotropic behavior and stabilizes the slurry for granule sedimentation effects. The mixture was set to rest for four hours to form the full thixotropy network. Before adding the WC–Co granules, 0.1 g/m^2^ of the dispersant Hypermer KD2 (Croda GmbH, Nettetal, Germany), based on the granules’ surface area, was added. For mixing, a laboratory mixer with a dissolver disc (BEVS 2501/1, BEVS Industrial Co., Ltd., Huangpu, China) was used. A total of 40 vol% of WC–Co granules, based on the monomer and cross-linker amount, were slowly added to the prepared resin during mixing. After the addition of the granules, the mixer speed was increased up to 4000 rpm for homogeneous slurry distribution. Subsequently, 28 mg of Butylated hydroxytoluene (BHT) (Sigma Aldrich Chemie GmbH, Taufkirchen, Germany) was added to prevent oxidation and free-radical formation. Finally, Bis(2,6-difluoro-3-(1-hydropyrrol-1-yl)phenyl)titanocene (Gelest, Inc., Morrisville, NC, USA) as a photoinitiator and an onium salt (H-Nu 254; Spectra Photopolymers, Millbury, MA, USA) as co-initiator were added in a mass ratio of 1.4:1 to the slurry during mixing. Degassing the slurry in a vacuum finalized the preparation.

### 2.3. Characterization of Thixotropic Behavior

A HAAKE™ RheoStress™ 1 rotational rheometer (Thermo Fisher Scientific, Karlsruhe, Germany) was used to characterize the rheological behavior. A plate-cone measuring system with a titanium cone with a 35 mm diameter and a cone angle of 2° was used. To determine the thixotropic slurry properties, a shear step with recovery was carried out as a combined oscillatory/rotational test with a given shear deformation according to [34]. Before the measurement, a pre-shear phase at 50 s^−1^ for 60 s and a rest time for 600 s was applied. For measurement, a deformation of 0.02% was applied as oscillation with ω = 10 rad/s in the linear viscoelastic range (LVR) for 300 s. Afterwards, a shear rate of 200 s^−1^ was applied for 10 s. Finally, a deformation of 0.02% as oscillation with ω = 10 rad/s was used for 500 s to evaluate the recovery. The complex viscosity was measured during the oscillatory measurement segments. The shear rate of 200 s^−1^ was chosen close to the applied shear rate during slurry recoating in vat photopolymerization.

### 2.4. Additive Manufacturing and Characterization of the WC–Co Green Body

Lithoz CeraFab 7500 (Lithoz GmbH, Vienna, Austria) was used to manufacture the WC–Co green body. The system provides a build space of 76.8 × 43.2 × 170 mm^3^ (X, Y, Z), and a lateral resolution of 40 μm (635 dpi; 1920 × 1080 pixels). In addition to the system, the resolution is determined by the volume fraction and size distribution of the particles used. The layer thickness can be varied in the range of 10–100 μm. A cuboid geometry of 6.2 × 6.2 × 4.8 mm^3^ (X, Y, Z) with curved edges was chosen as the specimen geometry. A layer thickness of 45 µm was set for printing. As exposure parameters, an intensity of 97.76 mW/cm^2^ and an exposure time of 30 s were defined. After printing, the green body was cleaned of uncured resin using compressed air. A metallographic sample was prepared perpendicular to the building direction in the middle of the specimen to identify defects. Quantitative microstructure analysis was used to determine the WC–Co granule/organic binder ratio in a defect-free region of the green body. The grayscale threshold was used for segmentation.

### 2.5. Debinding and Sintering

**Figure 2:**
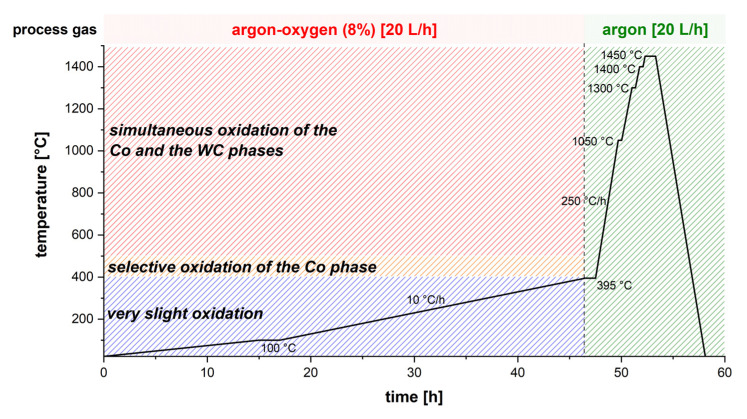
Temperature–time profile for debinding and sintering of the manufactured cemented carbide green bodies; oxidation ranges according to [36].

A thermogravimetric analysis was carried out to design a suitable debinding concept. Therefore, a green body of 23.6 mg was continuously heated up from 30 °C to 500 °C at a heating rate of 10 °C/h using the TGA/DSC 3+ HT thermal analyzer (Mettler–Toledo GmbH, Giessen, Germany). Argon with 8% oxygen at a constant gas flow of 3.0 L/h was used as the atmospheric gas. The heat treatment of the green body was performed in a gas-tight Al_2_O_3_ tube furnace HTRH 70–300, 1600 °C (GERO Hochtemperaturoefen GmbH & Co. KG, Neuhausen, Germany), with an inner diameter of 60 mm and length of 1000 mm. The green body was embedded in carbon black in an open-topped graphite crucible. For debinding, the sample was flushed with 20 L/h argon–oxygen (8%) up to 395 °C with a slow heating rate of 10 °C/h. During debinding, there was a continuous gas exchange and removal of the organic binder. The sample was flushed with pure argon for subsequent sintering. A temperature–time profile for sintering of cemented carbides was taken from the literature [35]. Isothermal steps were performed for solid-state sintering. The final liquid phase sintering was carried out at 1450 °C for 1 h. The temperature–time profile used is shown in Figure 2.

### 2.6. Characterization of the Sintered Specimen

The linear shrinkage of the sintered specimen was determined in the X, Y, Z direction. The surface quality was determined on the top as well as over the specimen side (building direction) by confocal microscopy (Smartproof 5, Carl Zeiss Microscopy GmbH, Oberkochen, Germany). An area of approximately 20 mm^2^ was used to measure the surface roughness parameters (Ra (arithmetical mean height), Rz (maximum height of profile)) and the surface texture parameters (Sa (arithmetical mean height), Sz (maximum height)) of the top surface. To evaluate the surface quality of the specimen side, an area of approximately 4 mm^2^ was measured. To evaluate the surface roughness parameters, the top surface was converted in a series of 9441 line profiles. The side surface was converted in a series consisting of 4067 line profiles. A metallographic sample was prepared to evaluate the WC grain FERET_max_ distribution after sintering and to determine the sinter density by quantitative microstructure analysis. The grayscale threshold was used for segmentation. This was performed by determining the porosity fraction of the sintered specimen and calculating the sinter density based on the theoretical material density of 14.3 g/cm^3^. In addition, five regions were defined to evaluate the porosity fraction perpendicular to the building direction in the middle of the specimen. LM and SEM analysis as well as X-ray diffraction was used to analyze the phases. For detection of the η-phase by LM, the sample was etched with Murakami’s reagent [37]. The X-ray diffraction patterns were collected with the SEIFERT Analytical XRD sun diffractometer (XRD Eigenmann GmbH, Schnaittach-Hormersdorf, Germany; formerly distributed by GE; Co radiation 50 kV, 35 mA). Three Meteor 1D linear detectors were used. Diffraction patterns were collected in Bragg–Brentano geometry with a measuring time of 600 s and a range from 30 to 130° in (2θ) with a step width of 0.013°. As mechanical parameters, Vickers hardness in HV10 and Palmqvist fracture toughness $W_{K}$ was measured by the Vickers indentation crack length approach according the Palmqvist method and was calculated by Equation (1) from Shetty et al. for cemented carbides [38,39].

(1) $$W_{K}=A\sqrt{H}\sqrt{\frac{P}{T}}$$

**Figure 3:**
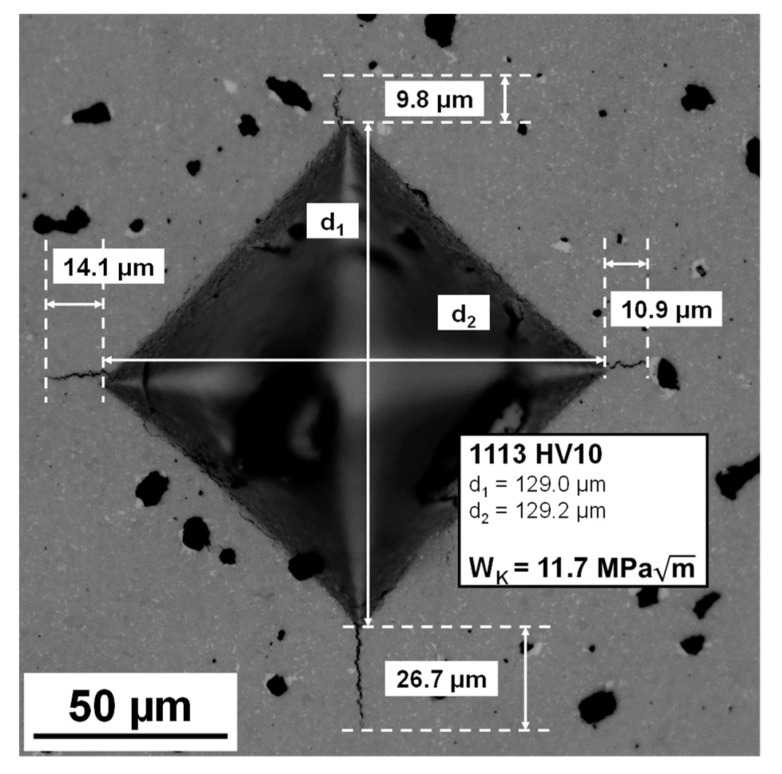
Vickers indentation for hardness and Palmqvist fracture toughness evaluation; LM, BF, 500×.

*A* is an empirical constant with a value of 0.0028, *H* is the hardness in N/mm^2^, *P* is the indent load and *T* is the total crack length in mm. For Vickers indentations with only three Palmqvist cracks or one crack ending in a pore, the average value of the existing cracks was assumed for the non-evaluable crack. If two formed cracks were not evaluable or if more than one crack was formed on a Vickers tip, the indentation was not measured and a new indentation was made. Figure 3 shows a representative example of an evaluated Vickers indentation.

## 3. Results and Discussion

### 3.1. Characterization of Raw Materials

**Figure 4:**
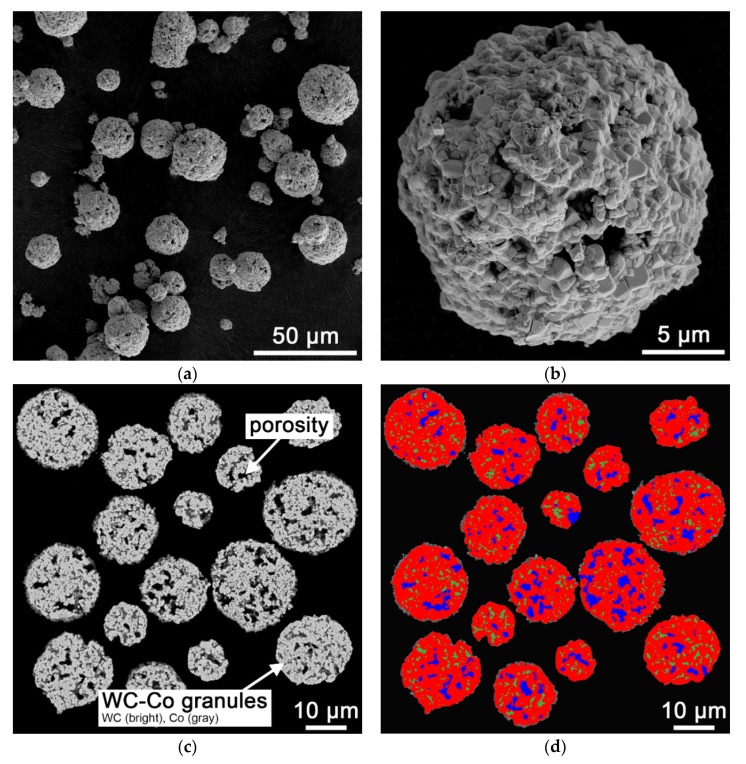
Starting WC–Co (88:12 wt%) granules; (**a**) overview image, SEM, SE, 500×; (**b**) detail image, SEM, SE, 4000×; (**c**) cross-section, SEM, AsB, 1000×; (**d**) quantitative analysis of WC fraction (red), cobalt fraction (green) and porosity fraction (blue).

Figure 4 shows an overview image (a), a detailed image (b), and the inner structure (c) of the WC–Co granules used. The granules show a spherical morphology with open porosity. Besides WC (bright) and Co (gray), an inner porosity (black) was detected. The false color image (Figure 4d) was used to determine the phase fractions and the porosity fraction.

Table 1 shows the d values of the granule size distribution determined by laser diffraction and the measured BET surface area. Also, the d values of the WC particle FERET_max_ distribution, the porosity fraction, and the WC/Co ratio determined by quantitative microstructure analysis are given in Table 1. 90% of the measured WC–Co granules are smaller than 24.3 µm. The measured WC particle FERET_max_ distribution corresponds to the fine-medium size range (0.8–2.5 µm) known for cemented carbides [40].

### 3.2. Thixotropic Slurry Properties

**Figure 5:**
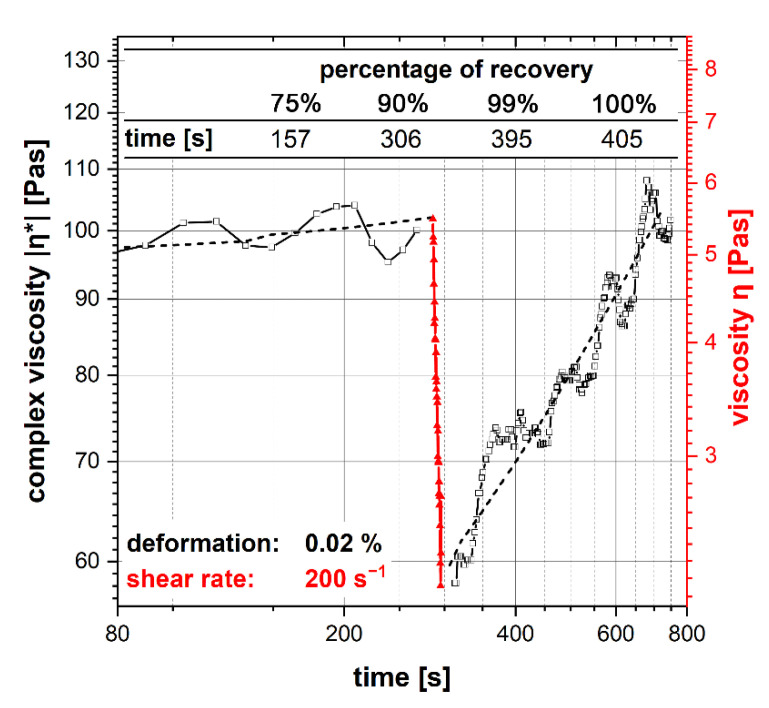
Shear step with recovery as combined oscillatory/rotational test with preset of shear deformation.

Figure 5 shows the result of the shear step with recovery as a combined oscillatory/rotational test with given shear deformation. The viscosity was reduced by about 57% by applying a shear rate of 200 s^−1^ for 10 s. After 157 s, 75% of the initial complex viscosity was recovered. The initial complex viscosity was reached again after 405 s.

The chosen thixotropic agent shows a shear-thinning behavior during squeezing to ensure a homogeneous coating. The result shows that the slurry can flow to squeeze out excess slurry and air bubbles between the building platform and the vat. When the shear stress is removed, the viscosity recovers to prevent sedimentation effects.

### 3.3. Characterization of the Green Body

**Figure 6:**
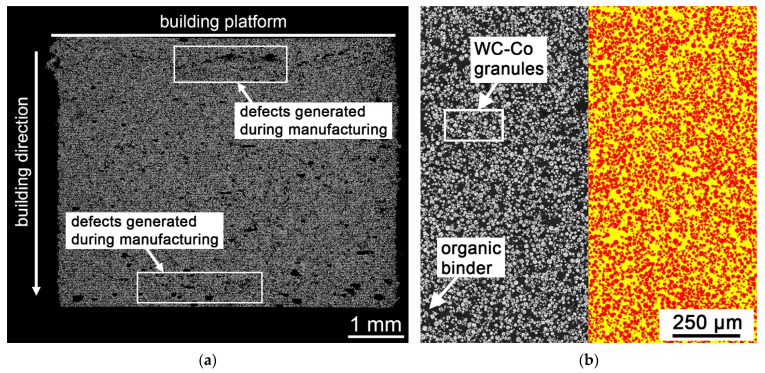
Evaluation of defects and WC–Co granule/organic binder ratio of the green body; (**a**) cross section of the green body, LM, BF, 500×; (**b**) evaluation using quantitative microstructure analysis, WC–Co granules (red), organic binder (yellow).

Figure 6 shows the cross section of a WC–Co green body perpendicular to the building direction in the middle of the specimen (a) and the quantitative analysis of the volume fraction of granules in a defect-free area (b). In addition to areas where the granules are closely packed, there are also areas where only a few granules are present. These defects can occur due to the high energy required to ensure successful photocuring. The adhesion forces between the newly cured layer and the PDMS coating of the vat increase strongly with increased exposure energies. These can lead to defects such as delamination of layers, holes, or adhesion of cured layers to the PDMS coating. Furthermore, insufficient rheological properties of the slurry can lead to defects [10]. Existing defects in the green body lead to an increased porosity fraction of the final sintered specimen [24,25,26].

The results of the quantitative analysis are in Table 2. The measurement shows that the true WC–Co granule/organic binder ratio of 0.67 matches the theoretical calculated ratio of 0.67 with a 40 vol% WC–Co granule fraction and a 60 vol% organic binder fraction.

**Figure 7:**
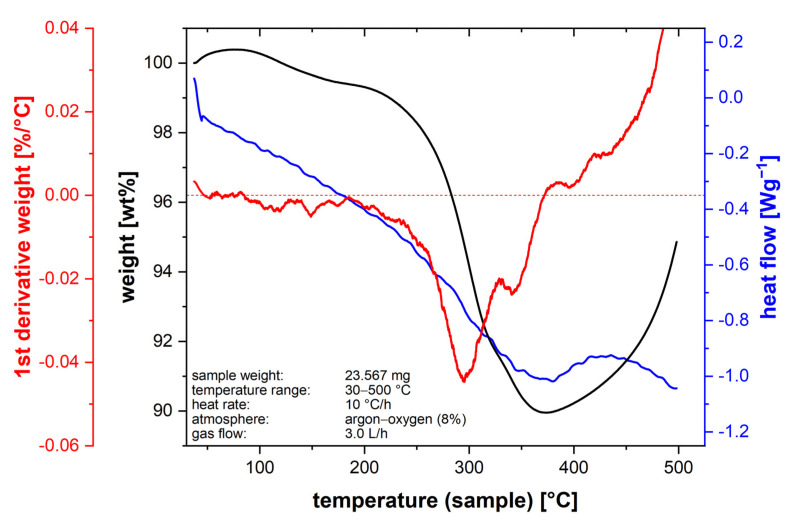
Thermogravimetric analysis of the WC-Co green body; weight loss (black), 1st derivative of weight loss (red), and heat flow (blue) as a function of temperature; Savitzky-Golay filtered.

Figure 7 shows the result of the thermogravimetric analysis. The black curve describes the weight loss of the green body as a function of temperature, while the 1st derivative of the weight loss is represented by the red curve. The blue curve outlines the heat flow as a function of temperature.

With increasing temperature, the binder was driven out of the green body. Under argon–oxygen (8%), the debinding was completed <400 °C. At temperatures <400 °C, only minor oxidation effects occur. Above a temperature of 400 °C, selective oxidation of the Co phase starts, followed by oxidation of the tungsten carbide phase >500 °C. [36] This also indicates the increase in weight >400 °C.

### 3.4. Characterization of the Sintered Specimen

**Figure 8:**
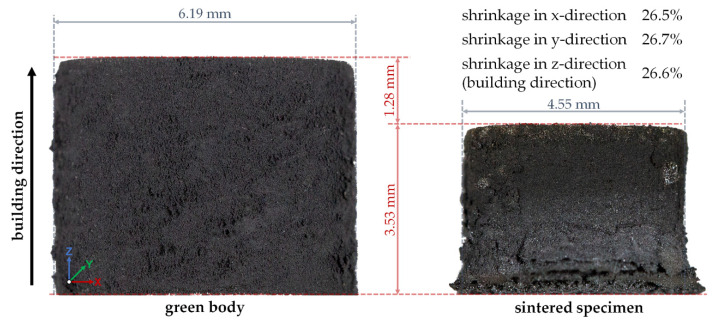
Side view of the green body and the sintered specimen.

Figure 8 shows a comparison of the green body and the sintered specimen. Due to the high organic binder fraction, a strong shrinkage was observed. The linear shrinkage during the heat treatment in X, Y, and Z dimensions of the sintered specimen in comparison to the manufactured green body can be taken from the side view images and varies between 26% and 27%. These linear shrinkages correspond to a volume shrinkage of approximately 60.5%.

**Figure 9:**
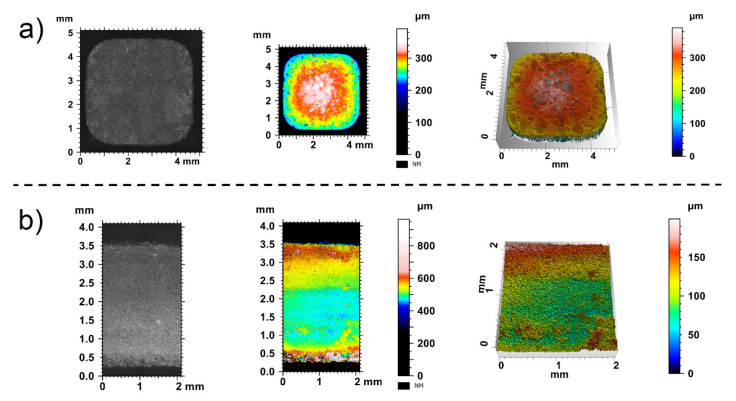
Surface roughness and texture measurement of the sintered specimen on the top (**a**) and of the specimen side (building direction) (**b**); true-color view, false color view, three-dimensional view.

The elephant foot formed at the bottom of the sintered specimen is attributed to the adhesion of the sintered specimen to the graphite plate during sintering. Figure 9 illustrates the surface roughness and texture measurement on the top (a) and on the side face (b) of the sintered specimen.

A slight convex geometry was observed on the top surface. Table 3 shows the determined roughness and texture parameters. The Ra and Rz values over the specimen side were approximately two times higher compared to the measured top surface.

The surface quality of Ra = 1.17 ± 0.18 µm was achieved for cemented carbides using MIM [41]. The Ra value achieved with vat photopolymerization is approximately 2 µm higher on the top surface and approximately 5 µm higher over the specimen side in the building direction. In comparison to other AM processes, such as material extrusion, material jetting, powder bed fusion, and sheet lamination, the measured surface quality lies in the same surface quality range [42,43,44]. Table 4 shows achievable surface qualities (Ra) with different additive manufacturing processes.

It should be mentioned that the achieved surface quality using additive manufacturing is strongly dependent on the type of powder and the process parameters used. In particular, the set layer thickness strongly determines the achievable surface quality [45].

**Figure 10:**
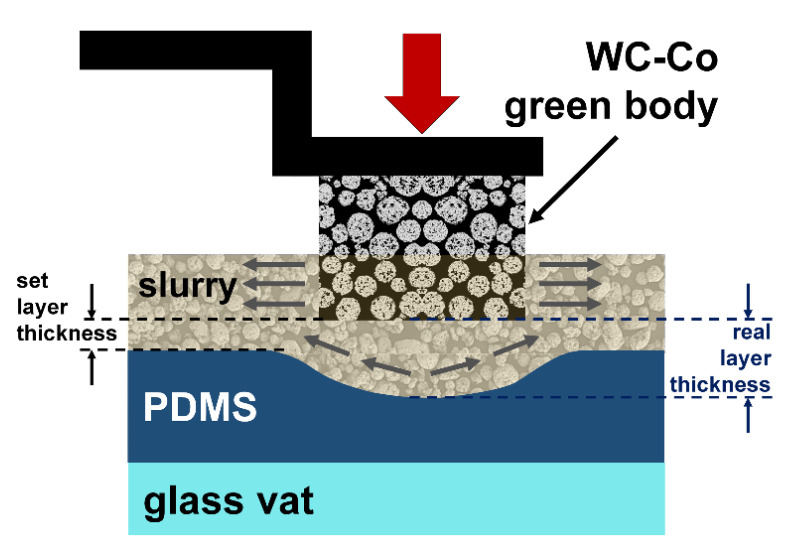
Elastic PDMS deformation during vat photopolymerization; own illustration, adapted from [46].

Ideally, additional slurry in the gap between the building platform and the PDMS-coated glass vat can flow out under the platform’s pressure, and a thin slurry layer of the set thickness will form. If the slurry cannot be driven out due to high viscosity, the PDMS coating elastically deforms. Figure 10 illustrates a deformed PDMS coating. Therefore, the final slurry layer thickness can be thicker than the set layer thickness and may lead to a convex geometry. The real layer thickness is also influenced by the PDMS layer thickness, slurry viscosity, coating blade height, and pressing speed [46].

**Figure 11:**
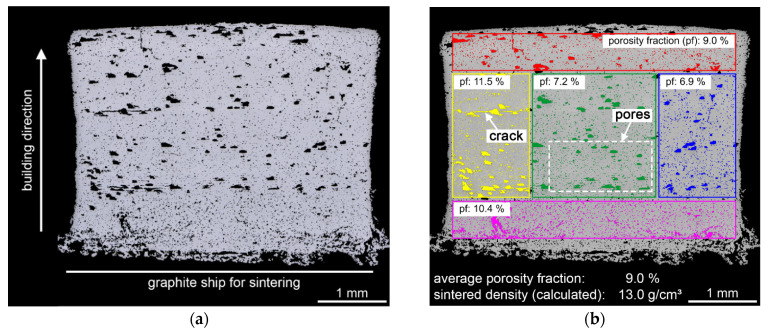
Microstructure of the sintered specimen; (**a**) overview image, LM, BF, 100×; (**b**) evaluation of the porosity fraction using quantitative microstructure analysis.

Figure 11 shows a light microscopy overview image perpendicular to the building direction in the middle of the sintered specimen (a) and the segmentation of the pores and cracks using quantitative microstructure analysis combined with grayscale threshold segmentation (b). An average porosity fraction of 9.0%, including the fraction of cracks, was measured. The smallest pore diameter measured was 1 µm, and the largest pore diameter was 167 µm. The detected cracks had a length between 63 µm and 449 µm. Compared to the inner area of the specimen, an increased porosity was observed in the area closed to the surface. Pores and cracks in the sintered specimen may be caused by defects in the green body or may occur during debinding. With the removal of the binder at the surface, vaporous binder components and degradation products move from inside the green body to the surface along with the softened binder under the action of pressure due to thermal expansion and capillary forces between the particles. Excessive pressure due to the increased heating can lead to cracks [47]. As a result, the sinter density of the specimen is 13.0 g/cm^3^ (theoretical material density: 14.3 g/cm^3^). The green body cannot be fixed during pressure-less sintering.

Compared to binder jetting cemented carbides, the achievable sinter density using vat photopolymerization was slightly lower. With binder jetting, 94% of the theoretical material density could be achieved. It was possible to achieve almost the theoretical material density of cemented carbides manufactured using binder jetting with subsequent post-compaction [14]. It should be mentioned that with material extrusion, material jetting, and conventional manufacturing methods, such as pressing and sintering or MIM, it is possible to manufacture components with an almost full theoretical material density (>99%) [5,13,15,48].

**Figure 12:**
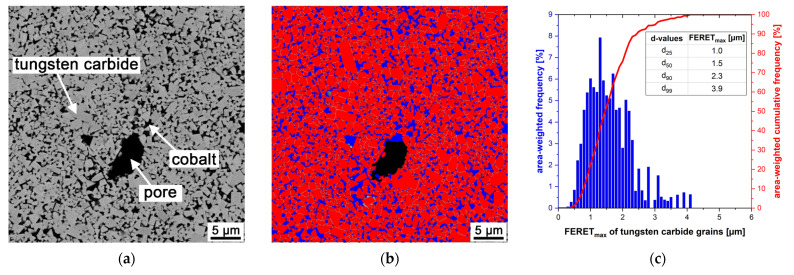
Evaluation of the WC grain FERET_max_ distribution; (**a**) representative image for the analysis, SEM, AsB, 2000×; (**b**) false color image generated by quantitative microstructure analysis, WC (red), Co (blue); (**c**) histogram of the tungsten carbide grains’ FERET_max_.

Figure 12 shows a SEM image of the sintered microstructure (a), the segmented image using quantitative microstructure analysis (b), and the evaluated WC grain FERET_max_ distribution (c). The WC grains are homogeneously distributed in the metallic cobalt phase.

Of the measured WC grains, 90% are smaller than 2.3 µm, and 99% of the measured WC grains are smaller than 3.9 µm. Compared to the starting WC grain FERET_max_ distribution, the d_90_ value increased by approximately 28%, the d_99_ value increased by approximately 63% during sintering. As a result, grain growth occurred and only 76% of the measured WC grains are in a size range which is suitable for metal cutting tool applications [31].

**Figure 13:**
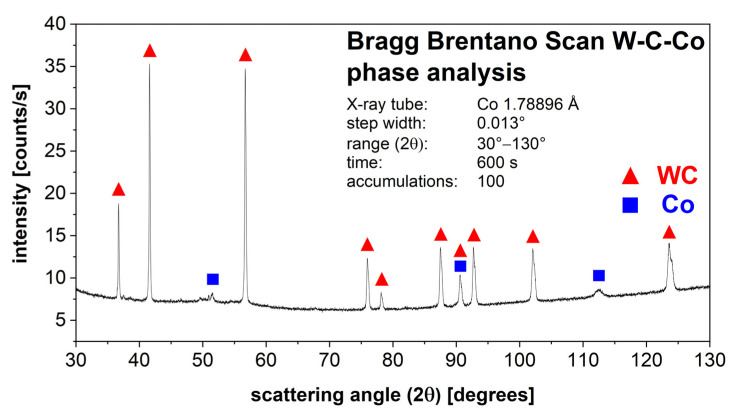
Sintered WC–Co (88:12 wt%) specimen shows third phase free X-ray diffraction spectrum.

Except for the top near-surface region (<500 µm depth), no η-phase was detected in the entire specimen by light microscopy and scanning electron microscopy. Free graphite was not detected in the whole sample. This result was also confirmed by X-ray diffraction analysis. Figure 13 shows the result of the performed phase analysis using X-ray diffraction. The spectrum shows only the presence of the tungsten carbide and cobalt phase peaks. A possible η-phase or free graphite formation is consequently smaller than the detection limit. This result shows that no significant de/carburization occurred during debinding and sintering. Impurities of the photoinitiator in the form of titanium and of the co-initiator in the form of antimony can be present in the sintered specimen [49].

Table 5 represents the measured Vickers hardness and Palmqvist fracture toughness values compared with literature data of pressed and sintered cemented carbides. The obtained hardness and Palmqvist fracture toughness values are lower than those for pressed and sintered WC–Co (88:12 wt%) components. The reduced mechanical properties can be attributed to the high porosity fraction and WC grain growth after sintering [31,50].

## 4. Conclusions

**Figure 14:**
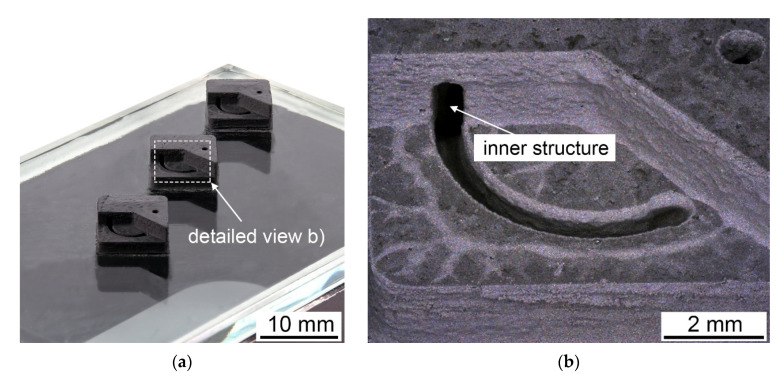
Prototype of a WC-12 Co (wt%) green body with inner structure, printed in an opened view; (**a**) overview image; (**b**) detailed view of the inner structure.

This study shows that vat photopolymerization can be used to manufacture cemented carbide green bodies. The slurry also enables the manufacturing of complex geometries. An opened, printed prototype with an inner structure is shown in Figure 14. This shows that inner cooling structures, which are of particular importance for tools, can also be printed. The smallest printed feature was a bore with a diameter of approx. 900 µm. The limitation was the cleaning of uncured slurry in cavities.

The final resolution of vat photopolymerization combined with the developed WC–Co slurry is influenced by the furnace technology and the selected temperature–time profile during debinding and sintering. Only if the exact temperature–time profile can be controlled and a precise furnace atmosphere can be ensured, is it possible to manufacture specimens with specified properties. Applying a shear rate of 200 s^−1^ for 10 s reduced the viscosity by 57%. This creates a homogeneous coating of the slurry in the vat. A total of 75% of the thixotropic structure was recovered after 157 s, and the full initial complex viscosity was achieved after 405 s. Consequently, the slurry was stabilized for sedimentation effects at rest. However, the green bodies manufactured exhibit defects in the form of pores and fine cracks. The rheological properties, as well as the exposure parameters, showed a significant influence on the formation of defects [10]. Also, excess slurry that could not flow out of the gap between the building platform and the PDMS-coated vat due to high viscosity led to elastic deformation of the PDMS layer and a slightly convex print form over the specimen height [46]. Based on the results of thermogravimetric analysis, it was possible to design a temperature–time profile for debinding. Debinding was performed with a slow heating rate of 10 °C/h up to 395 °C. Due to the strong oxidation effects of the Co binder phase, debinding had to be completed below 400 °C [36]. After sintering, a linear shrinkage of 26–27% was observed. This corresponds to a volume shrinkage of approximately 60.5 vol%, which matches with the added organic binder fraction of 60 vol%. The specimen exhibited a Ra value <7 µm and a Rz value <43 µm, a Sa value <27 µm and a Sz value <400 µm. Due to the low solid fraction of 40 vol% in the slurry, the microstructure exhibits local defects and a porosity fraction of 9.0%. Because of the high porosity fraction, the calculated sinter density was 1.3 g/cm^3^ smaller than the theoretical material density of 14.3 g/cm^3^ [48]. Grain growth occurred during sintering. The d_99_ value of the WC grains’ FERET_max_ increased by approximately 63% during the heat treatment compared to the initial WC particle FERET_max_ in the WC–Co granules. Except for the top near-surface region, no η-phase was detected by LM and SEM examination. X-ray diffraction also showed only the presence of the WC and Co phase. Impurities of the photoinitiator and the co-initiator can be present in the sample. The present pores and cracks in the sintered specimen significantly limit the mechanical material properties. Compared to pressed and sintered components with the same Co fraction and WC grain size, the Vickers hardness was approximately 16% smaller and the Palmqvist fracture toughness was approximately 27% smaller [48]. A defect-free and pore-free microstructure are necessary to achieve the high mechanical cemented carbide properties required for metal-cutting tool applications.

In an initial proof of concept experiment, a subsequent post-Sinter HIP (overpressure sintering) was investigated. Based on an increased sinter density, the hardness of the manufactured samples could be increased by approx. 21% (1395 ± 23 HV10), and the Palmqvist fracture toughness could be increased by 25% (15 ± 1 $\mathrm{MPa}\sqrt{m}$) in an η-phase free region. Defects at the outer areas of the sample and the formation of the η-phase in the whole sample were still present. In further studies, hot isostatic pressing (HIP) and Sinter-HIP (overpressure sintering) will be investigated to minimize the residual porosity fraction and defects.

## Figures and Tables

**Table 1:** Size distribution, BET surface area, porosity fraction, and WC/Co ratio of the used granules.

	d_10_ [µm]	d_50_ [µm]	d_90_ [µm]	d_99_ [µm]	BET [m^2^/g]	Porosity Fraction [%]	WC/Co Ratio
WC–Co (88:12 wt%) granules	8.4	15.8	24.3	32.4	0.13	9.1 ± 3.5 ^b^	8.4 ± 1.9 ^b^
Initial FERET_max_ of WC particles ^a^	0.7	1.1	1.8	2.4			

^a^ FERET_max_ of WC particles measured with quantitative microstructure analysis. ^b^ Error range related to the evaluated granules in Figure 4d.

**Table 2:** Quantitative evaluation of the granule fraction and organic binder fraction.

	Fraction of WC–Co Granules ^a^ [%]	True Fraction of WC–Co Granules ^b^ [%]	Fraction of Organic Binder ^a^ [%]	True WC–Co Granule/ Organic Binder Ratio ^a^
WC–Co (88:12 wt%) green body solid fraction: 40 vol%	42.5 ± 1.2	38.6 ± 1.2	57.5 ± 1.2	0.67

^a^ Average value of two evaluated green bodies. ^b^ Minus 9.1% inner granule porosity.

**Table 3:** Surface roughness/texture parameters of the sintered top surface and of the specimen side.

	Ra [µm]	Rz [µm]	Sa [µm]	Sz [µm]
surface roughness/texture parameters of the top surface	3.1 ^a^	19.8 ^a^	26.6	391
surface roughness/texture parameters of the specimen side (building direction)	6.2 ^b^	42.6 ^b^	23.2	199

^a^ The measured profile series includes 9441 line profiles. ^b^ The measured profile series includes 4067 line profiles.

**Table 4:** Achievable surface quality with different additive manufacturing methods [42,43,44].

	Ra [µm]
vat photopolymerization (with developed WC–Co slurry)	3.1–6.2
vat photopolymerization (especially for stereolithography)	2–40
powder bed fusion (especially for selective laser sintering)	5–35
material extrusion (especially for fused deposition modelling)	9–40
material jetting	3–30
sheet lamination (especially for laminated object manufacturing)	6–27

**Table 5:** Measured hardness and Palmqvist fracture toughness compared with literature data.

	Hardness [HV10]	Fracture Toughness $K_{\mathrm{IC}}/W_{K}\;[\mathrm{MPa}\sqrt{m}]$
WC–Co (88:12 wt%) literature data [48]	~1375 ^a^	~16–17
WC–Co (88:12 wt%) measured after sintering	1157 ± 59 ^b^	12 ± 2 ^c^

^a^ Literature data measured in HV30. ^b^ Average value of minimum 15 hardness measurement points. ^c^ Measured with the Palmqvist method for cemented carbides.
